# Can we get the benefits of integrated services? An evaluation of the delivery of integrated prenatal HIV, syphilis and hepatitis B testing services in China

**DOI:** 10.1186/1472-6963-14-S2-P140

**Published:** 2014-07-07

**Authors:** Jianhong Xia, Yuanzhu Ma, Shuang Gao, Shannon Rutherford, Xiaozhuang Zhang, Cordia Chu

**Affiliations:** 1 Centre for Environment and Population Health, Griffith University, Brisbane, Queensland, Australia; 2 Health Care Department, Guangdong Province Maternal and Children Health Care Hospital, Guangzhou, Guangdong, China

## Background

Integration of services for prevention of mother-to-child transmission (PMTCT) of HIV into routine maternal and child health care has been promoted as a priority strategy by WHO to help optimize health outcomes for mother and children [1]. However, integrated services require a shift in the management model from providing stand-alone services to integrating services, which requires more complex management [2]. China initiated the process of integrating prenatal HIV, syphilis and hepatitis B testing services in 2009, and many health agencies need to work together [3]. So this study is to evaluate the effectiveness of the current integrated services and examine barriers to coordination in this complicated service system in China.

## Materials and methods

The research was conducted in Guangdong province, used mixed quantitative and qualitative methods. We drew quantitative data from routine monitoring system for PMTCT and a quantitative survey; and collected interviews for qualitative data to assess and examine barriers.

## Results

The testing rates of prenatal HIV, syphilis and hepatitis testing were 95%, 47% and 47% respectively, which were inconsistent, although the three testing services have been integrated. For the prenatal HIV testing service, it took an average of one month to get results because of multi-agency referrals. In addition, almost 80% of the positive mothers were not referred to the hospitals for continuous monitoring and treatment services. The reasons behind these statistics are that the responsibilities assigned to the different health agencies were not clear, and outcome evaluation was not consistent among different health agencies. There was a lack of concrete coordination and referral scheme which must be built on the basis of effective collaboration and communication between different health agencies.

## Conclusions

The results indicate that the benefits of integrated services have not been completely achieved in China. The many barriers to coordination in a complex health service system form a great challenge to effective delivery of integrated services. A concrete operational strategy is needed to fill the gap in order to achieve the desirable outcomes from integrated services.

## References

[B1] WHO Global health sector strategy on HIV/AIDS 2011-2015 2011: a new health sector agenda for HIV/AIDS 2011 Geneva, Switzerland: World Health Organization http://www.who.int/hiv/pub/advocacy_brochure/en/pdf

[B2] Dichinson C Attawell K Druce N Progress on scaling up integrated services for sexual and reproductive health and HIV Bull World Health Organ 2009 14 846 851 10.2471/BLT.08.059279 20072770 PMC2770277

[B3] MOH Report on Women and Children’s Health Development in China 2011 2011 Beijing, China: Ministry Of Health http://www.gov.cn/gzdt/2011-09/21/content_1952953.htm.pdf

